# Evaluating the Effectiveness, Safety, and Satisfaction Rates of Phenol 90%, Trichloroacetic Acid 100%, and Radiofrequency in Lateral Matricectomy for the Treatment of Ingrown Toenails: A Triple‐Arm Clinical Trial

**DOI:** 10.1002/hsr2.70326

**Published:** 2025-01-08

**Authors:** Mohammadreza Ghassemi, Zahra Keshavarz, Elham Behrangi, Afsaneh Sadeghzadeh Bazargan, Alireza Jafarzadeh, Azadeh Goodarzi

**Affiliations:** ^1^ Department of Dermatology, Rasool Akram Medical Complex Clinical Research Development Center (RCRDC), School of Medicine Iran University of Medical Sciences (IUMS) Tehran Iran

**Keywords:** clinical trial, ingrown toenail, lateral matricectomy, nail surgery, onychocryptosis

## Abstract

**Background and Aims:**

This study aimed to evaluate the effectiveness, safety, and satisfaction rates of three different lateral matricectomy methods for treating ingrown toenails: 90% phenol, 100% trichloroacetic acid (TCA), and radiofrequency (RF) ablation. Our objective was to identify which method offers superior outcomes regarding postoperative pain, healing time, aesthetic results, and complication rates.

**Methods:**

Conducted between August 2022 and June 2023, the study included 12 eligible patients divided into three groups: Group 1 underwent lateral matricectomy with 90% phenol, Group 2 with 100% TCA, and Group 3 with RF treatment. Preoperative assessments were conducted, and patients were followed up on the day after surgery and at Weeks 1, 2, and 3, as well as at Months 1, 2, 6, and 9 postoperatively (totaling nine assessments). Pain levels were measured using the Visual Analog Scale (VAS), while wound discharge and bleeding were rated on specified scales.

**Results:**

Mean pre‐ and postoperative (second day) pain scores were 6.0 ± 3.3 and 0.75 ± 0.67 for Group 1, 6.25 ± 4.92 and 1.75 ± 0.58 for Group 2, and 6.25 ± 2.25 and 3.50 ± 0.50 for Group 3, with significant differences observed. RF treatment showed higher moderate postoperative pain compared to mild pain in the phenol and TCA groups. Average healing time was 22.75 ± 3.50 days (phenol), 40 ± 12.91 days (TCA), and 26.25 ± 6.70 days (RF), with phenol demonstrating faster healing, although differences were statistically insignificant. The median aesthetic outcomes favored phenol with a score of 5.0.

**Conclusion:**

Lateral matricectomy with 90% phenol, 100% TCA, and RF all provide unique advantages for ingrown toenail treatment. Phenol exhibited high success rates, minimal complications, faster healing, and excellent aesthetic outcomes.

**Trial Registration:**

IRCT, IRCT20220228054152N1. Registered 03 January 2023, https://www.irct.behdasht.gov.ir/trial/65907.

## Summary

- **What Is Already Known About This Topic?**
  - An ingrown toenail is a painful condition where the edge of the nail plate grows into the surrounding skin, known as the paronychium. It can result in significant discomfort and disability for patients.
  - Various treatments have been proposed for ingrown toenails, all focusing on removing the edge of the nail plate from the paronychium and altering the lateral matrix.
  - Several methods, including the use of radiofrequency, trichloroacetic acid, and phenol for lateral matricectomy, have been proposed in previous studies. Each method has been associated with a certain level of recovery and satisfaction.
- **What Does This Study Add?**
  - On the second day postoperation, pain scores were significantly lower for phenol and TCA treatments compared to RF treatment. The phenol and TCA groups experienced mild postoperative pain, while the RF group reported higher, but still moderate, pain levels.
  - The average healing times for the phenol group, TCA group, and RF group were 22.75 ± 3.50, 40 ± 12.91, and 26.25 ± 6.70 days, respectively. This suggests faster healing with phenol; however, the differences in healing time were statistically insignificant.
  - Both phenol and RF treatments yielded the highest aesthetic results, with median and mean scores of 5.0 (8.75) and 4.5 (7.50), respectively. In contrast, the TCA group achieved a decent yet notably less impressive aesthetic outcome, with median and mean scores of 3.5 (3.25).

**Abbreviations**
RF: radiofrequency
TC: trichloroacetic acid
VAS: visual analog scale

## 1 Introduction

An ingrown toenail, also known as onychocryptosis, is a prevalent and often painful condition that occurs when the edge of the nail plate penetrates the paronychium [1]. In response to this trauma, the affected skin produces highly vascular granulation tissue, which gradually protrudes beyond the nail plate. Concurrently, the skin becomes susceptible to microbial invasion, leading to inflammation, infection, and potentially cellulitis [2, 3]. These complications can significantly disrupt daily activities and diminish the quality of life for those affected [4].

Various treatment methods have been formulated for onychocryptosis, tailored to its stage and severity. Stage 1 (inflammation) is characterized by edema, erythema, nail fold swelling, and pain upon pressure. Stage 2 (acute infection) is marked by increased edema, pronounced erythema, seropurulent drainage, infection, and nail fold ulceration. The most severe form, Stage 3 (granulation), presents with extensive drainage, chronic infection, granulation tissue formation, and hypertrophy of the lateral nail wall [5]. Conservative methods are typically effective for Stages 1 and 2 [2, 6], while partial avulsion of the nail and nail matrix (with or without cauterization) is recommended for Stage 3, Stage 2, and recurrent Stage 1 cases [5, 7, 8]. It is essential to note that failure to perform a matricectomy increases the likelihood of recurrence [9].

Chemical cauterization options include phenol, trichloroacetic acid (TCA), and sodium hydroxide [2, 4]. Phenol induces coagulation necrosis, effectively cauterizing both the nail matrix and the surrounding soft tissues [10]. However, this often results in excessive drainage and delayed healing [11]. In contrast, TCA triggers coagulation necrosis through protein denaturation without significant systemic toxicity, reducing the risk of postoperative drainage and infection. Notably, it self‐neutralizes following epidermal and dermal necrosis [11, 12]. However, evidence regarding TCA's long‐term efficacy remains limited [2, 12].

Mechanical matricectomy techniques, such as carbon dioxide lasers or radiofrequency (RF) ablation, provide greater selectivity and efficacy in targeting the matrix horn compared to chemical methods, albeit at a higher cost and increased technical complexity [4, 13, 14].

Currently, no consensus exists regarding the optimal treatment option that balances functional and aesthetic outcomes, side effects, and rates of recurrence. Although numerous studies have investigated the efficacy of individual [11, 15, 16] or paired [17, 18, 19] treatment modalities, very few have conducted comparative analyses of three distinct methods, particularly concerning postoperative outcomes.

In light of the considerable clinical importance and varying outcomes associated with different methods of matricectomy, this study initially aimed to comprehensively compare three techniques: phenol 90%, TCA 100%, and RF. However, recognizing the pivotal role of phenol as a widely accepted gold standard in the treatment of ingrown toenails, we further analyzed the comparative effectiveness of phenol and TCA. This focused comparison will elucidate the nuances between the two methods, enhancing our understanding of their respective merits and limitations.

## 2 Materials and Methods

### 2.1 Patients

Our study was conducted from August 2022 to June 2023. Twelve patients meeting the inclusion criteria were divided into three groups: Group 1 underwent lateral matricectomy with 90% phenol, Group 2 with 100% TCA, and Group 3 with RF treatment. Among the patients, seven had symptoms for 6–12 months, and five had symptoms for over 1 year. At the initial visit, three patients exhibited site infections (one in each group), leading to a postponement of surgery to allow for infection treatment. All patients had previously received conservative treatments at other clinics with no response.

### 2.2 Inclusion Criteria

1. Patients diagnosed with Stage 2 or 3 onychocryptosis.
2. Age range: 18 to 65 years.
3. Patients must have a history of symptoms lasting at least 6 months.
4. Individuals who had not responded to conservative treatments before the study participation.

### 2.3 Exclusion Criteria

1. Patients with structural nail disorders (e.g., nail dystrophies, severe deformities).
2. Individuals with significant peripheral arterial disease affecting circulation.
3. Patients diagnosed with diabetes mellitus or other comorbidities that could impair healing.
4. Individuals with foreign metal bodies (e.g., pacemakers or metal implants).
5. Patients who have undergone previous lateral matricectomy.
6. Patients with active infections at the treatment site.
7. Individuals with conditions known to affect wound healing (e.g., immunocompromised states, chronic skin conditions).

### 2.4 Randomization and Blinding

In this clinical trial, randomization was performed to ensure that each patient had an equal chance of being assigned to any of the three treatment groups (Group 1: phenol 90%, Group 2: TCA 100%, Group 3: RF). A computer‐generated random number sequence was used to allocate the participants to their respective groups at the time of recruitment. This method of randomization minimizes selection bias and helps to produce comparable groups with respect to demographic and clinical characteristics.

To further ensure the integrity of the study, blinding was implemented where feasible. Patients were blinded to the specific treatment they received, as they were informed only that they would be undergoing a procedure for their ingrown toenail without specifics about the method. The surgeons performing the procedures were not blinded to the treatment allocation due to the nature of the interventions; however, postoperative assessments were conducted by a separate clinician, blinded to the group assignments. This helped to avoid bias in evaluating outcomes such as pain, wound discharge, and aesthetic results.

Additionally, the use of standardized assessment tools allowed for objective measurement of outcomes. By employing both randomization and blinding, the study aimed to enhance the validity of the results, ensuring that any differences observed among the treatment groups were attributable to the interventions themselves rather than preexisting biases.

### 2.5 Ethical Approval

The study received ethical approval from the Iran University of Medical Sciences, with the ethics code number IR.IUMS.FMD.REC.1400.310, to ensure compliance with ethical standards. Informed consent was obtained from all participants before their inclusion in the study.

### 2.6 Intervention Methods

The surgical procedure comprised several steps [6]. Initially, digital nerve block anesthesia was achieved using 2% adrenaline‐free lidocaine. The area was prepared with drapery, and a tourniquet was applied to the toe to prevent bleeding. The affected nail plate was detached from the nail bed on the ingrown side using septum elevators, and the ingrown edge was longitudinally sliced 3–4 mm away from the onychocryptotic side, followed by the complete removal of the nail edge. Subsequently, the nail bed and matrix were partially excised by supraperiosteal stripping over the phalanx. Any granulation, crust, or hypertrophic tissue present was excised from the lateral nail fold.

For Groups 1 and 2, 90% phenol or 100% TCA was applied three times for 1 min each (a total of 3 min) [4, 7]. Afterward, the operation site was rinsed with a saline solution to prevent excessive damage to the surrounding areas. In Group 3, the lateral horn of the matrix was ablated using RF. The RF electrode was applied three times for 5 s each (total of 15 s) [1]. Following this, the tourniquet was released, and an antibiotic‐containing ointment was administered. The treated nail was covered with a gauze bandage. Patients were discharged 30 min after surgery and advised to elevate and rest their foot for the remainder of the day, with acetaminophen as an option for pain relief. Dressings were changed after 2 days and subsequently daily until no discharge was observed at the wound site.

### 2.7 Assessment Method

**Figure 1:**
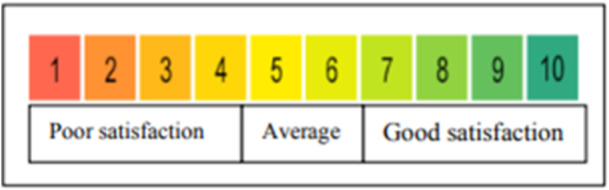
Grade scale to evaluate patient's satisfaction.

Patients were examined preoperatively and followed up on the next day, as well as in the first, second, and third weeks, and first, second, sixth, and ninth months postoperatively (9 assessments in total). Pain levels were assessed using the Visual Analog Scale (VAS) [12]. Wound discharge (rated from 0 for *lack of discharge* to 4 for *high discharge*), bleeding (rated from 1 for *low* to 3 for *high*), and cosmetic satisfaction (rated from 1 for *very low* to 10 for *very high*) were evaluated using “Grade scale to evaluate patient's satisfaction” [16] (Figure 1).

Healing time was determined as the duration required for complete re‐epithelialization of the nail bed and regression of edema. Recurrence and wound infection were assessed through yes/no questions.

### 2.8 Data Analysis

Statistical analyses were performed using R and SPSS 23, with a significance level set at *p* < 0.05. Data distribution was assessed using the one‐sample Kolmogorov–Smirnov test. Intergroup differences in continuous data related to postoperative pain and healing time were evaluated using MANOVA and/or ANOVA tests. For categorical data on wound discharge, bleeding, and cosmetic outcomes, the Kruskal–Wallis test was utilized, while the maximum likelihood method was applied for dichotomous data on wound infection.

Prespecified analyses included comparisons of primary outcomes such as pain and healing time among groups, whereas exploratory analyses encompassed subgroup analyses based on the duration of symptoms: 6–12 months versus over 1 year. Both one‐sided and two‐sided tests were employed as appropriate for the analyses.

By adhering to the SAMPL guidelines for statistical reporting, we ensured clarity and precision in presenting our findings. Statistical terms and abbreviations were defined as follows: [DEFINE TERMS/ABBREVIATIONS].

## 3 Results

The mean age of the patients (18 to 65 years old) was 33.9 years (SD = 17.42). A total of 83.3% of the patients were male, while 16.7% were female. The right big toe was affected in 66.7% of the patients. At their first visit, 58.3% reported having symptoms for less than 1 year. The majority of patients were in Stage 2 (83.3%), with 16.7% in Stage 3. The study groups did not differ significantly from one another in terms of demographic information.

Mean prior and postoperative (second day) pain scores were as follows:

- Group 1 (phenol treatment): Preoperative 6.0 ± 3.3; Postoperative 0.75 ± 0.67
- Group 2 (TCA treatment): Preoperative 6.25 ± 4.92; Postoperative 1.75 ± 0.58
- Group 3 (RF treatment): Preoperative 6.25 ± 2.25; Postoperative 3.50 ± 0.50

Multivariate analysis of variance (MANOVA) revealed significant differences in pre‐ and postoperative pain across treatment groups (Pillai's trace = 0.766, *F* = 14.71, *p* < 0.001, *η*² = 0.770). Follow‐up analysis of variance (ANOVA) indicated that the severity of postoperative pain on the second day was significantly higher in the RF group, while the phenol and TCA groups experienced mild pain (Table 1). It is important to note that pain lasted approximately 1 week for the phenol and TCA groups, but extended to 2 weeks for the RF group.

**Table 1:** Comparison of postoperative pain and healing time.

Variable	Treatment method			*F*	Sig.
	Phenol	TCA	RF		
Pain	0.75^a^	1.75^a^	3.50^b^	15.50	0.001
Healing time	22.75^a^	40.00^b^	26.25^ab^	4.458	0.045

*In each row, means followed by dissimilar letters differ significantly (*p* < 0.05 for the Tukey test).

The average healing time for the groups was:

- Group 1 (phenol): 22.75 ± 3.50 days
- Group 2 (TCA): 40 ± 12.91 days
- Group 3 (RF): 26.25 ± 6.70 days

Although phenol treatment was associated with faster healing, the differences in healing time were statistically insignificant (*p* > 0.99).

No patient experienced recurrence, but postoperative wound infection was observed (Table 2). At the first visit, one patient from each group suffered from chronic inflammation and infection in their nails. One patient from the phenol group and two from the TCA group reported wound infections during the second and third follow‐ups, respectively. No inflammation or infection was observed in Group 3. Maximum likelihood estimation revealed significantly lower infection rates for the RF group during the first and second postoperative weeks. The TCA group showed the highest rate of postoperative infection (Table 2).

**Table 2:** Number of patients with wound infections.

	Prior intervention	Second day	First week	Second week
Phenol	1	0	1	1
TCA	1	0	2	2
RF	1	0	0	0
*p*‐value	1.00	1.00	0.021	0.021

According to Table 3, at the first postoperative visit (on the second day), mild discharge was observed in 75% of patients in the phenol group, while moderate discharge was seen in 25% of this group. Among the phenol group, 75% showed improvement in wound discharge after one or 2 weeks. In contrast, the TCA group showed moderate discharge in 50% of patients, with 50% experiencing high discharge, leading to prolonged wound discharge. In this TCA group, 25% of patients had a reduction in discharge after 2 weeks, and 75% experienced improvement after 3 weeks. The RF group exhibited mild wound discharge, with patients becoming discharge‐free within the first week. Consequently, it can be concluded that TCA resulted in longer and more significant wound discharge compared to the other treatments. Bleeding during and after the operation was statistically similar across all groups (*p* > 0.99).

**Table 3:** Comparison of postoperative wound discharge, bleeding, and aesthetic outcomes.

		Method	Mean rank	*χ* ^2^	*df*	Exact Sig.
Wound discharge	Second day	Phenol	5.25	8.556	2	0.018
		TCA	10.25			
		RF	4.00			
	First week	Phenol	5.25	8.556	2	0.018
		TCA	10.25			
		RF	4.00			
Bleeding	During surgical operation	Phenol	4.13	4.660	2	0.113
		TCA	9.25			
		RF	6.13			
	Postsurgical operation	Phenol	4.50	4.400	2	0.212
		TCA	9.00			
		RF	6.00			
Aesthetic outcome		Phenol	8.75	5.971	2	0.051
		TCA	3.25			
		RF	7.50			

**Figure 2:**
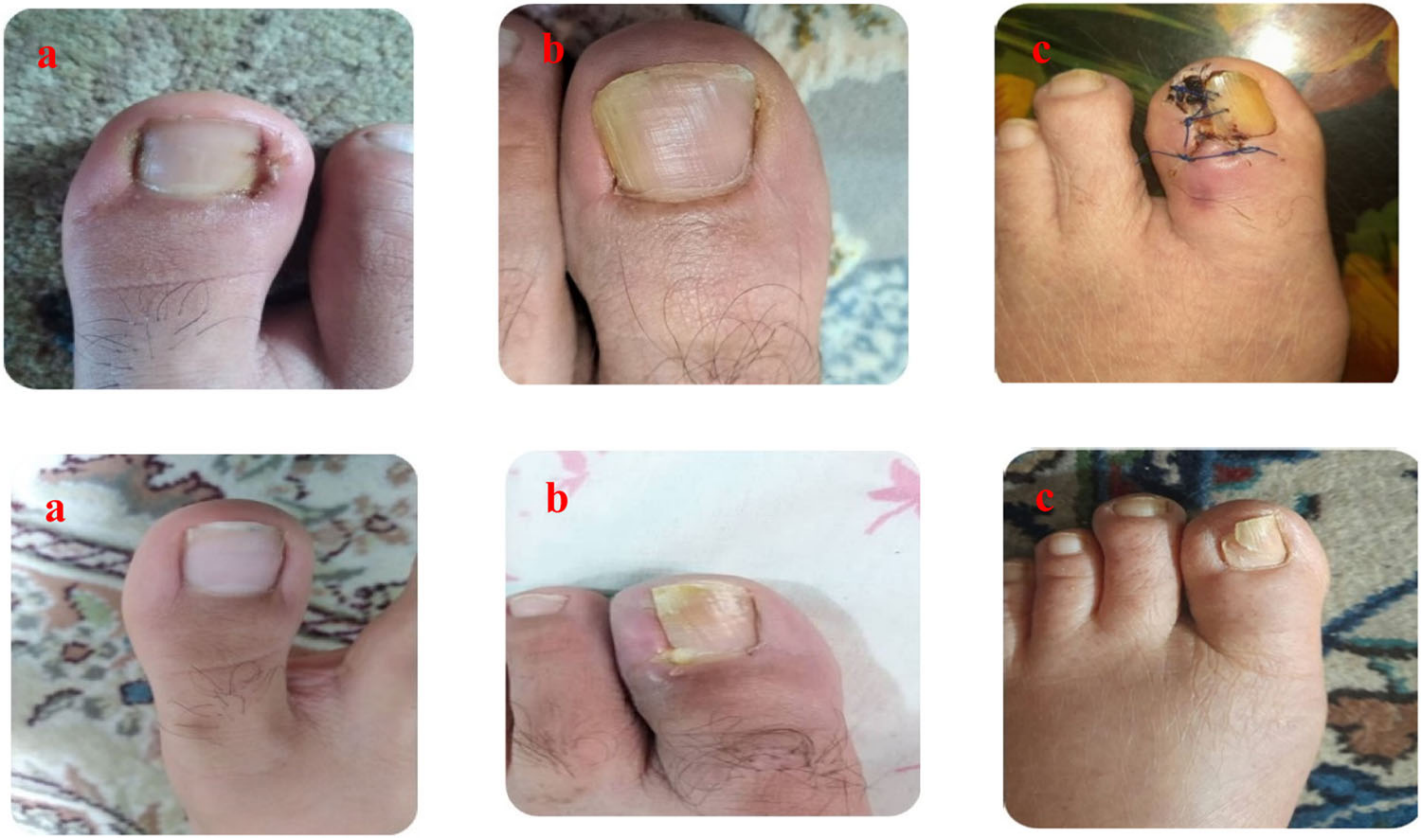
(a) Nice aesthetic results with no recurrence at 9 months after surgery in the phenol group. (b) Wound infection in the TCA group 1 week after surgery. (c) Mild discharge 2 days after surgery in the RF group, with no recurrence at 9 months after surgery.

The median and mean of aesthetic outcomes were reported as follows: phenol group (median 5.0, mean 8.75), RF group (median 4.5, mean 7.50), and TCA group (median 3.5, mean 3.25) (Figure 2 and Table 3). These results indicate the most favorable aesthetic outcomes for the phenol and RF treatments, while TCA yielded an acceptable yet significantly less satisfying aesthetic outcome.

## 4 Discussion

This study reinforces the consensus in existing literature that phenol remains a superior choice for treating ingrown toenails, particularly concerning safety, effectiveness, and patient satisfaction compared to TCA. Previous research consistently demonstrates that phenol therapy not only reduces recurrence rates but also minimizes postoperative complications, aligning with our findings.

When considering phenol, one of its standout advantages is its effectiveness in preventing the recurrence of ingrown toenails by destroying the nail matrix at the origin of the problem. The procedure is generally straightforward and tends to involve only minor discomfort for patients. However, a significant drawback is the potential for chemical burns or irritation to surrounding tissue if not applied carefully. Furthermore, there is a risk of complications such as infection or delayed healing, which should be weighed against its benefits [3, 4].

In contrast, TCA serves as an effective chemical agent for treating ingrown toenails by cauterizing the affected area. It allows for a quick procedure with minimal bleeding, making it an attractive option for some practitioners. Nevertheless, TCA is not without its downsides. The risk of tissue damage due to aggressive application and variability in patient responses can pose challenges. Additionally, patients treated with TCA may experience a higher potential for recurrence of the ingrown nail [11].

RF treatment offers another alternative, with its ability to precisely target the affected area, helping to decrease damage to surrounding tissues and lowering the risk of infection. This method generally results in less postoperative pain compared to traditional surgical approaches. However, it is important to consider the potential disadvantages of RF treatment, including its higher cost, limited availability, and the requirement for specialized equipment and training, which could restrict access for some patients [6, 8].

In this study, we examined patients aged 18 to 65 years, primarily young adults in the second and third stages of ingrown toenails, employing three treatment methods: phenol, TCA, and RF. The demographic profile of our cases mirrors those in studies by Ahsan et al. [17] and Moustaide et al. [14], suggesting that the active lifestyle of young adults may contribute to the prevalence of ingrown toenails. Moreover, the male‐to‐female ratio in our cohort was higher than usual, consistent with previous observations [15, 20]. Notably, 66.6% of cases involved ingrown toenails on the right big toe, corroborating findings from Ahsan et al. [17] and Tabowei et al. [21].

Over a 9‐month follow‐up, we observed no recurrence of ingrown toenails across all treatment methods, suggesting the efficacy of lateral matricectomy utilizing phenol, TCA, and RF. This is significant, given that prior studies report recurrence rates ranging from 1.7% to 29% for surgical treatments [22]. Our results indicate that both chemical and mechanical cauterization can effectively reduce recurrence [11, 23, 24].

Our findings indicate a statistically significant difference in the effectiveness of the three methods for reducing wound infection and inflammation. The RF group demonstrated the lowest wound infection rates, paralleling the findings of Kim et al. [24]. While Barreiros et al. [23] and Terzi et al. [11] reported low infection rates post‐TCA treatment, our results indicated that phenol significantly reduced postoperative infections compared to TCA, likely due to its antiseptic and antibacterial properties, as noted by Ahsan et al. [17].

Furthermore, our results highlighted average healing times of 22.75 days for the phenol group, 40 days for TCA, and 26.25 days for RF, which align with recovery timeframes of 14 to 28 days reported in various studies [15, 25]. Notably, patients treated with phenol exhibited significantly faster healing compared to those treated with TCA, consistent with outcomes from Misiak et al. [18] and Moustaide et al. [14]. While healing times between the phenol and RF groups, and the TCA and RF groups were statistically similar, this contrasts with findings by Kwon et al. [16].

Satisfaction with aesthetic outcomes was notably high among patients treated with phenol and RF, whereas the TCA group reported less satisfactory results, corroborating the observations made by Kim et al. [24]. This contrasts with Moustaide et al. [14], who found comparable cosmetic outcomes between phenol and TCA treatments.

Finally, we also assessed postoperative pain, with moderate pain noted in the RF group and mild pain levels for phenol and TCA treatments. This finding is consistent with the work of Ahsan et al. [17] and Moustaide et al. [14]. The higher VAS scores for RF ablation can be attributed to more aggressive tissue excision. Discharge levels were highest in the TCA group, while mild postoperative discharge for phenol and RF patients generally resolved within 7 to 14 days. In contrast, the moderate to high discharge levels observed in the TCA group improved within 14 to 21 days, aligning with observations from Barreiros et al. [23] and Terzi et al. [11].

Overall, this comprehensive analysis underscores the benefits of phenol treatment over TCA and highlights the potential of RF as a complementary method, presenting valuable insights for clinical practice in managing ingrown toenails.

### 4.1 Limitations

This study is limited by its small sample size of 12 patients, which restricts the generalizability of the findings. The modest cohort size, while providing initial insights into the efficacy, safety, and satisfaction rates of phenol (90%), TCA (100%), and RF in lateral matrixectomy, necessitates caution in interpretation. To establish more definitive conclusions, future research with larger sample sizes is essential. Increasing the number of participants will enhance the robustness and reliability of the results obtained in this study.

## 5 Conclusions

In summary, the findings suggest that while both TCA and RF methods offer certain advantages in treating onychocryptosis, phenol remains the gold standard, particularly when considering its long‐term efficacy and superior outcomes. Lateral matricectomy using 90% phenol is particularly noteworthy for its high success rate, minimal postoperative complications, and excellent aesthetic results, alongside features such as zero recurrence and fast healing times. Although TCA and RF also demonstrate low recurrence and satisfactory results, phenol consistently outperforms them in key areas, including pain management and overall patient satisfaction. To further clarify the distinctions and optimize treatment protocols, larger‐scale and longitudinal studies focusing specifically on phenol's efficacy and long‐term outcomes are recommended. This will enhance our understanding and guide clinicians in selecting the most effective approaches for managing onychocryptosis.

## Author Contributions

Contributions to the current study includes M.G. and Z.K. and A.S. and E.B. in study idea and design and in the literature review, and drafting and revising the manuscript critically for importance intellectual content. A.J. and Z.K. in conducting the trial, data gathering, drafting the proposal, following up with ethical committee for approval, and revising the manuscript critically for importance intellectual content. M.G. in drafting the revised manuscript and literature review, and analysis and interpretation of revised version and drafting the manuscript. M.G. in the proposal preparation and statistics and analysis and drafting the revised manuscript. M.G. and A.G. in the study supervision, data gathering and literature review, and drafting the manuscript and both are corresponding authors. All authors have read and approved the final version of the manuscript. The corresponding author, Azadeh Goodarzi, had full access to all the data in this study and takes complete responsibility for the integrity of the data and the accuracy of the data analysis.

## Ethics Statement

All information obtained from patients was kept confidential and evaluated anonymously. All patients studied adhered to the Helsinki ethical principles and the study protocol was registered at Iranian Registry of Clinical Trials with code: IRCT20220228054152N1, Registration date: 2022‐01‐03, URL: https://www.irct.behdasht.gov.ir/trial/65907. This project was approved by the Ethics Committee of Iran University of Medical Sciences with the title: “Evaluating the Effectiveness, Safety, and Satisfaction Rates of Phenol 90%, Trichloroacetic Acid 100%, and Radiofrequency in Lateral Matricectomy for the Treatment of Ingrown Toenails: A Triple‐Arm Clinical Trial”, with the ethical code IR.IUMS.FMD.REC.1400.310, date of approval: 2021‐05‐31. The patients signed informed consent for participating in the study.

## Consent

The authors have nothing to report.

## Conflicts of Interest

The authors declare no conflicts of interest.

## Transparency Statement

The lead author Zahra Keshavarz, Azadeh Goodarzi affirms that this manuscript is an honest, accurate, and transparent account of the study being reported; that no important aspects of the study have been omitted; and that any discrepancies from the study as planned (and, if relevant, registered) have been explained.

## Data Availability

The data that support the findings of this study are available from the corresponding author upon reasonable request.
